# Readmissions on Teaching Versus Non-Teaching Services: Are They Any Different?

**DOI:** 10.7759/cureus.8529

**Published:** 2020-06-09

**Authors:** Jawaid A Shaw, Asghar Ali, Rabia Qaiser, Erynn Layman, Cynthia Fagan, Owen Schwartz, Adam Sima, Monica Hazelrigg

**Affiliations:** 1 Internal Medicine, Virginia Commonwealth University, Richmond, USA; 2 Internal Medicine/Hospital Medicine, Hunter Holmes McGuire VA Medical Center/Virginia Commonwealth University, Richmond, USA; 3 Internal Medicine, Hunter Holmes McGuire VA Medical Center, Richmond, USA; 4 Internal Medicine/Hospital Medicine, MedStar Washington Hospital Center, Washington, DC, USA; 5 Biostatistics, Virginia Commonwealth University School of Medicine, Richmond, USA

**Keywords:** readmissions, teaching service, non-teaching service, veteran affairs, comparisons

## Abstract

Introduction

There is a paucity of comparative data on readmissions between teaching services (TS) and nonteaching services (NTS). Therefore, we designed this study to determine if there are any differences in readmissions between the two services.

Materials and methods

A unique cohort of 384 readmissions during one year was retrospectively examined at Hunter Holmes McGuire Veterans Medical Center. The data on patient demographics, baseline characteristics, comorbid illnesses, length of stay (LOS), and reasons for readmission within 30 days were extracted.

Results

There were no differences in readmission rates (8.2% vs. 10.2%; P = .135), LOS during index admission (4.2 ± 4.8 vs. 4.1 ± 3.5; P = .712), and age-adjusted Charlson Comorbid Index Score (6.1 ± 3.0 vs. 6.8 ± 2.8; P = .037) between the TS and NTS groups. However, the reasons for readmissions between the two groups were statistically significantly different (P < .01). Specifically, these differences were found between system issues and new diagnoses. The NTS showed higher rates of readmissions secondary to new diagnoses and systems issues, whereas the TS showed higher rates of secondary to clinician issues and disease progression.

Conclusions

We have a new understanding of the difference in reasons for readmissions between TS and NTS; it possibly results from the different structures of the two teams, which may help us address readmissions in a different light to improve overall readmission rate.

## Introduction

The admission of a patient to the hospital is not a simple phenomenon as it involves robust inpatient care, discharge planning, and arrangement for an appropriate transition of care. Any compromise to this chain of events while delivering inpatient healthcare may result in poor patient outcomes and avoidable readmissions. We recognize the significant negative impact of readmissions on healthcare resources; there is a national priority directed towards preventing readmissions to hospitals [1]. An earlier study revealed that nearly 20% of Medicare beneficiaries discharged from hospitals were readmitted within 30 days, resulting in estimated losses of 17.4 billion dollars for 2004 [2]. Hence, any decrease in readmission rates would be a welcome step. In a survey of the literature, we find that some of the readmissions can stem from clinician issues, hospital issues, patient issues, disease progression, or from a combination of factors [3,4]. We attempted to compare the differences in readmissions between teaching and nonteaching services to understand the reasons for these and to devise methods to decrease the readmissions. In academic settings, the clinical care team staffed by trainee physicians, along with supervising attending physicians, is called the teaching service (TS), whereas clinical care teams without trainee physicians are known as the nonteaching service (NTS). We retrospectively studied the causes of readmission between TS and NTS groups to test our hypotheses that these readmissions might be different secondary to the difference in the structure of these two teams.

## Materials and methods

The study was conducted at Hunter Holmes McGuire VA Medical Center (VAMC), which is a teaching affiliate of Virginia Commonwealth University, both at Richmond, Virginia. After Institutional Review Board approval, we conducted a retrospective chart review of all readmissions within the 30 days of index hospitalization-discharge from general medicine service from April 1, 2009, to March 30, 2010. Readmission to the hospital was defined as any patient readmitted to the hospital within the 30 days of index discharge. The readmission was considered as a sentinel event, and this led to a review of the index admission-discharge to ascertain the reason for this readmission while taking into account the reason for the current admission (readmission) as well. We compared readmissions between the TS and NTS to ascertain differences in readmission outcomes. The determination of whether the readmission belonged to the TS or the NTS was made from the electronic health record and further verified at the time of physical chart review to perform a correct assignment. The inclusion criteria were a) patients readmitted within 30 days of index admission-discharge during the study period and b) only one readmission per discharge and at the first occurrence during the study period was counted. The exclusion criteria were a) planned readmissions for any reason and b) readmission to the surgical specialties.

At the study hospital, the structures of the TS and NTS teams are as follows. The TS is composed of a supervising attending, an upper-level resident, two to three interns, and two to three medical students who work together as a unit. There are four such TS teams and one NTS team. The medicine hospitalist attending, along with the midlevel providers without any trainee staff involvement, run the NTS. The majority of the time, the same attending physicians (hospitalists) work back and forth on the TS and NTS. However, at times the medical subspecialists staffed the TS for supervision. At the study site, during the night and off-hours, the TS was led by an in-house upper-level resident with no direct attending supervision, but the attending was available on a pager for guidance. In contrast, the NTS had a fully staffed nighttime physician. The hospital bed controller, in a sequential fashion, performed patient assignment to the TS and NTS. The only exception to this rule occurred if individual TS were at the maximum allowed number of patients that it can manage, the so-called “cap” in-compliance with Accreditation Council for Graduate Medical Education rules. If such is the case, that TS gets skipped, and the new admission goes to the next team in the sequence. However, per hospital policy, the NTS does not get capped and is open for admissions at all times. We categorized readmissions into six categories: 1) clinician issues (e.g., the current standard of care/guidelines not followed, relevant data points ignored by a physician), 2) system issues (e.g., poor execution of discharge planning, lack of setting up of outpatient follow-up), 3) patient issues (e.g., non-compliance with medications, not showing up for follow-up appointments), 4) disease progression (e.g., the progression of a neoplastic process, the progression of decompensated liver disease, 5) new diagnosis (e.g., patient discharged after chest pain evaluation getting admitted with gastrointestinal bleeding and the like), and 6) combination of factors (i.e., more than one of the above causes leading to readmission). Two hospital medicine attending physicians assigned the readmission to one of the above categories. If there was any conflict between the two investigators in such an assignment, that case was referred to as the principal investigator for final adjudication and consensus. The two investigators had interrater reliability (kappa) of 0.76 (standard error [SE] = 0.03).

Patient characteristics collected for the index admission included age, gender, race, age adjusted Charlson comorbidity index (CMI) parameters to calculate CMI score and hospital severity index score to see if the two services were comparable for patient acuity [5]. Others included the number of medications/supplies at the index admission and discharge, code status of the patient, attending physician experience in years (calculated from the time of initial board certification) and if the post-discharge follow-up contact was established or not, which is usually done telephonically within 48 hours of discharge by the case manager. We examined the number of days to readmission from index discharge, length of stay (LOS) at index admission, and reasons for readmission. The origination destination of patients at index admission and disposition of patients at index admission and readmission were recorded.

The data were summarized using descriptive statistics. Patient characteristics by service status TS and NTS were compared by equal variance, two-sample t-tests, and Pearson’s chi-square tests. Unequal variance two-sample t-test and Fisher’s exact test were used where appropriate. Multivariable logistic regression was used to assess the relationship between discharge disposition in readmission patients returning to home with age, sex, race, number of comorbid conditions, number of medications prior to admission and first discharge, code status, attending physician experience in years, post-discharge call and reasons for readmission. All tests were performed at the 0.05 significance level using SAS V9.4 (Cary, NC).

## Results

Over the study period, there were a total of 4,316 admissions to the inpatient medicine service, 3,474 (78%) admissions were to TS, and 842 (22%) admits to NTS. Overall, 384 patients qualifying for our inclusion and exclusion criteria enrolled in the study. The TS had 298 (8.6%) and NTS 86 (10.2%) readmissions (p > 0.05), respectively. The majority of patients were male (96%) with no differences in age between TS and NTS group (67.5 ± 12.2 years vs. 69.1 ± 12.2 years; P = .269). All the patient characteristics, except the attending physician experience in years, failed to demonstrate a significant statistical difference between the TS and NTS groups. Physicians in the TS group had more experience than in the NTS group (15.2 ± 11.1 years vs. 5.6 ± 8.1 years; P < .001). Table 1 summarizes the patient characteristics at the index admission-discharge. There was no statistical difference between the number of bed days for TS and NTS on index admission (4.2 ± 4.8 days vs. 4.1 ± 3.5 days; P = .712) and on readmission (4.4 ± 4.1 days vs. 4.7 ± 4.3 days; P = .521), respectively. Table 2 shows the average bed days between the services. However, the reasons for readmissions differed significantly between the two groups (P < .01). Patients in the NTS group had a higher incidence of readmissions because of system issues and new diagnoses, while the TS group had a higher incidence of readmissions because of disease progression and clinician issues. Table 3 summarizes the reasons for readmissions.

**Table 1:** Patient characteristics on index admission-discharge 1. N = 371, 2. N = 383, 3. N = 330, 4. N = 258 ^a^ Fisher’s exact test. ^b^ Chi-Square test. ^c^ Two-sample t-test (pooled). ^d^ Two-sample t-test (Satterthwaite).

Characteristics	Overall (N = 384)	Teaching service (N = 298)	Nonteaching service (N = 86)	P-value
Age (years)	67.8 (12.2)	67.5 (12.2)	69.1 (12.2)	.269^c^
Gender				
Men	368 (96%)	285 (96%)	83 (97%)	>.999^a^
Women	16 (4%)	13 (4%)	3 (3%)	
Race				
Caucasian	222 (58%)	174 (58%)	48 (56%)	.665^a^
African American	159 (41%)	122 (41%)	37 (43%)	
Hispanic	0 (0%)	0 (0%)	0 (0%)	
Other	3 (1%)	2 (1%)	1 (1%)	
Charlson-Comorbidity Index Score	6.3 (2.9)	6.1 (3.0)	6.8 (2.8)	.037
Number of medications prior to admission^2^				
0-10 medications	102 (27%)	84 (29%)	18 (21%)	.126^a^
11-20 medications	180 (47%)	140 (47%)	40 (47%)	
21-30 medications	80 (21%)	55 (18%)	25 (29%)	
31+ medications	21 (5%)	18 (6%)	3 (3%)	
Code status^3^				
Full code	275 (83%)	216 (86%)	9 (83%)	.586^b^
Do not resuscitate	55 (17%)	45 (14%)	10 (17%)	
Attending physician experience (years)^2^	13.1 (11.2)	15.2 (11.1)	5.6 (8.1)	<.001^d^
Number of medications at first discharge				
0-10 medications	67 (18%)	54 (19%)	13 (15%)	.246^b^
11-20 medications	193 (50%)	154 (51%)	39 (45%)	
21-30 medications	93 (24%)	65 (22%)	28 (33%)	
31+ medications	30 (8%)	24 (8%)	6 (7%)	
Post-discharge call				
Yes	278 (72%)	215 (72%)	63 (73%)	.840^b^
No	106 (28%)	83 (28%)	23 (27%)	
Hospital Severity Index Score^4^				
<2.5%	130 (50%)	108 (53%)	22 (41%)	.275^b^
2.5-5%	55 (21%)	41 (20%)	14 (26%)	
5-10%	39 (15%)	30 (15%)	9 (17%)	
10-30%	28 (11%)	22 (11%)	6 (11%)	
>30%	6 (2%)	3 (1%)	3 (6%)	N

**Table 2:** Average bed days between service groups NTS: Nonteaching service; TS: Teaching service; SD: Standard deviation. ^a^ Satterthwaite method. ^b^ Pooled method (instead, use equal variance unless specified)

Characteristic	N (%)	Average Bed Days (SD)	P-values
Index admission			
TS	298 (78%)	4.2 (4.8)	.712^a^
NTS	86 (22%)	4.1 (3.5)	
Readmission			
TS	298 (78%)	4.4 (4.1)	.521^b^
NTS	86 (22%)	4.7 (4.3)	
Reason for readmissions			
Clinician Issues			
TS	17 (100%)	6.3 (5.1)	-
NTS	0 (0%)	0 (0)	
System Issues			
TS	5 (45%)	2.4 (2.1)	.409^b^
NTS	6 (55%)	3.7 (2.7)	
Patient Issues			
TS	23 (89%)	3.1 (2.2)	.689^b^
NTS	3 (11%)	3.7 (0.6)	
Disease Progression			
TS	146 (83%)	4.7 (4.6)	.562^b^
NTS	29 (27%)	5.3 (4.5)	
New Diagnosis			
TS	65 (66%)	3.8 (3.5)	.647^a^
NTS	33 (34%)	4.2 (5.0)	
Combination of factors			
TS	42 (74%)	4.3 (3.7)	.278^b^
NTS	15 (26%)	5.5 (3.0)	

**Table 3:** Reasons for readmission between teaching service and nonteaching service

Readmission Reason	Teaching service (N = 298)	Nonteaching service (N = 86)
Clinician Issues	17 (6%)	0 (0%)
System Issues	5 (2%)	6 (7%)
Patient Issues	23 (8%)	3 (3%)
Disease Progression	146 (49%)	29 (34%)
New Diagnosis	65 (22%)	33 (38%)
Combination of Factors	42 (14%)	15 (17%)

Patients resided at home prior to the index admission (95%), with an assisted living facility (1%), skilled nursing facility (2%), and a nursing home (2%) accounting for the remainder. Overall, there were no differences in residence status of the patients in the TS and NTS groups prior to index admission (p = 0.526), post index admission discharge (p = 0.600), and post readmission discharge (P = .786). There was a total of 20 (5%) deaths or hospice placement at readmission with 15 (6%) in the TS group and five (5%) in the NTS group (P > .05). This indicates that patient residence at admission and post-hospital discharge disposition was comparable.

Logistic regression was conducted to assess the relationship in readmission discharge disposition in patients returning to home between TS and NTS groups taking into account patient and readmission characteristics. Age, attending physician experience, and post-discharge calls were found to be related to discharge disposition. A one-year increase in patient age increased the odds of going to a higher level of residential care or hospice care (odds ratio [OR] = 1.06). Likewise, a one-year increase in physician experience increased odds of going to a higher level of residential care or hospice care by 3% (OR = 1.03). Table 4 shows logistic modeling assessing the relationship between discharge disposition in readmission patients with patient and readmission characteristics.

**Table 4:** Logistic model assessing the relationship between discharge disposition in readmission patients with patient and readmission characteristics CMI: Comorbidity index

Characteristic	Levels	Odds Ratio	95% Confidence Interval	P-values
Service group	Teaching service vs. Nonteaching service	1.14	(0.45, 2.90)	.778
Age		0.94	(0.91, 0.97)	< .001
Gender	Men vs. Women	4.29	(1.03, 17.8)	.045
Race	White vs. non-White	0.67	(0.33, 1.36)	.268
CMI Score		0.90	(0.80, 1.02)	.095
Number of medications prior to admission		1.00	(0.92, 1.09)	.962
Number of medications at first discharge		1.02	(0.94, 1.11)	.624
Code status	Full code vs. No resuscitation	1.56	(0.70, 3.49)	.280
Attending physician experience (years)		0.96	(0.93, 0.99)	.006
Post-discharge call	Received call vs. no call	3.57	(1.82, 6.99)	< .001
Reasons for readmission	Clinician vs. combination of factors	6.31	(0.91, 43.6)	.166
	System vs. combination of factors	5.55	(0.48, 63.7)	.341
	Patient vs. combination of factors	0.61	(0.14, 2.69)	.043
	Disease progression vs. combination of factors	1.57	(0.63, 3.94)	.398
	New diagnosis vs. combination of factors	2.68	(0.93, 7.79)	.579

## Discussion

Our study shows that there are no differences between readmission rates, LOS at the index admission, and overall mortality at readmission between the two services. However, as far as the analysis of reasons for readmission is concerned, NTS had higher rates of new diagnoses and systems issues, whereas TS had higher rates of clinician and disease progression issues. We believe that systems issues for NTS might have resulted from a lack of robust discharge planning practices, which might have included unclear patient communications and poor attention to transitions of care for this group. We are fully cognizant of the fact that it is not possible to avert all readmissions, especially if secondary to entirely different or a new diagnosis, which is not connected to the index admission diagnoses and progression of diseases, and the like. However, in the NTS group, there were no clinician issues, which may reflect a more direct involvement of the attending physicians, in comparison to 17 (5.7%) in the TS group, which could be secondary to indirect involvement of the attending physicians on TS. The higher incidence of disease progression in TS (50%) may as well point towards the similar indirect involvement of the teaching attending physicians on TS during the index admission as well as the intrinsic progression of the disease.

Hospital readmissions are burdensome with negative financial implications both for patients and the health care systems. However, it is difficult to uniformly ascertain readmissions with a wide range of 5% to 79% (median 27.1%) having been put forth [6]. In our cohort, the overall readmission rate for the whole group was around 9% and within this range. Upon review of literature, mixed results, meaning both increase and decrease in the LOS at the index admission between NTS and TS, were revealed [7-10]. In our study, we did not find a statistically significant difference in the LOS between TS and NTS as well, which probably reflects similar access and utilization of resources at the index admission by the two groups. In our data, the all-cause mortality was not statistically significant between TS and NTS (5% vs. 6%). These results align with other studies comparing patient outcomes between teaching and nonteaching medical services [8-10]. Again, a majority of readmitted patients (73%) were on more than ten medications, which goes well with the results of another study where polypharmacy has been suggested to be one of the factors associated with readmissions [11]. The high number of medicines and supplies at discharge may be a reflection of the multiple comorbidities and older age group of our cohort. The attending physician experience in years between the two groups was found to be significant in our study, explained by the fact that TS was manned at times by medical subspecialists who were older while as most of the hospitalist attending physicians are in a younger age group. In our study, a one-year increase in physician experience increased odds of going to higher residential care or hospice care by 3% (OR = 1.03). We noted that physicians with greater than 11 years of experience had overall higher readmissions (40.6%) and average LOS of 4.4 days. Previous studies have also shown that as physician experience increases, it may lead to an increase in mortality and the LOS for the attending physicians belonging to subspecialties [12,13].

Balla et al. reported medication discrepancy (44%), deficient workup (33%), brief hospital stay (31%), error in diagnosis (16%), and overlooking important laboratory findings (12%) during the index admission as the causes of readmission [14]. In an earlier study, Frankl et al. concluded that previously diagnosed medical conditions accounted for 75% of readmissions, which is nearly comparable to our study [4]. To be able to avoid some of the readmissions, various prediction models if applied at the time of index admission may be able to forecast who the patients with higher chances of getting readmitted are and thus, potentially mitigate some readmissions by helping to optimize patient care and direct appropriate resources at this subgroup of patients earlier on [15,16]. We feel that early post-hospital discharge primary care follow-up may help reduce readmission rates, as demonstrated by a decrease in 30-day readmission rate for congestive heart failure patients [17]. While there is no single intervention that can reduce readmissions, multimodality approaches may be effective [18]. It may be beneficial to design a curriculum for trainees to educate them about optimal discharge planning, broadening their understanding to help them see who the subset of patients at increased risk of readmission are, and practice safer transition of care. As TS and NTS have basic differences in the composition of teams, the responsibility of team members, workflow, and functional processes, this could explain the differences in the causes of readmissions between the two services.

The limitation of our study is that it represents a single-center retrospective experience with a select cohort of male veteran patients, thus making it difficult to generalize the results. The hospitalist attending physicians who worked both on TS and NTS performed data extraction, and all the investigators were trained by the PI prior to the start of the study to ensure uniformity in data collection and to minimize unintentional bias. The investigators could not be blinded as the data was extracted using a computerized system where it is evident as to what service the patient was assigned to on admission from signature blocks and note titles. However, all necessary measures were taken to minimize any bias, including adjudication at times, by the PI. Readmissions happening outside the VA system may not have been captured, leading to some data losses as well.

## Conclusions

Overall, there are no significant differences in readmission rates, length of stay at the index admission, and readmission, along with no difference in mortality at readmission between the TS and the NTS groups. However, the reasons for readmissions are different between TS and NTS. This could be secondary to the difference in the intrinsic structure of the two services, possibly leading to a difference in the level of supervision and workflow patterns between the two services. Our findings shed new light, highlighting the differences in the causes of readmissions between the two services and open up a new area of opportunity to address these readmissions in light of these differences. This may pave the way for a reduction in overall readmissions for both services. In the future, a larger study to look further into whether this phenomenon holds true may need to be conducted.
